# Sex-specific psychological mechanisms of cyber reactive aggression: evidence from Chinese college students

**DOI:** 10.3389/fpsyg.2026.1764030

**Published:** 2026-03-17

**Authors:** Qian-Nan Ruan, Fei-Rui Ni, Ru-Ying Yu, Yongjian Lin

**Affiliations:** 1 Wenzhou Depression Specialty Center, Wenzhou Seventh People's Hospital, Wenzhou Medical University, Wenzhou, China; 2 Department of Student Affairs, Wenzhou University, Wenzhou, China

**Keywords:** cyber reactive aggression (CRA), hostile attribution bias (HAB), network analysis, revenge motivation (RM), sex differences, trait anger (TA)

## Abstract

This study investigated sex-specific psychological mechanisms underlying cyber reactive aggression (CRA) among Chinese college students, addressing a critical gap in understanding how trait anger (TA), hostile attribution bias (HAB), and revenge motivation (RM) differently influence male and female online aggressive behaviors. Using convenience sampling, we collected data from 926 students (371 males, 555 females) across 12 universities in China. Network analysis with mgm package revealed distinct sex-specific patterns: male participants showed strong TA-HAB and RM-CRA associations, while female participants exhibited prominent HAB-CRA relationships. Notably, maternal education levels were positively associated with aggressive behavior in both sexes (males: weight = 0.22; females: weight = 0.14), while left-behind experiences uniquely was associated with higher HAB in males (weight = 0.55). Multi-group structural equation modeling further validated these sex-specific pathways: TA was significantly associated with CRA in both sexes (males: β = 0.40, *p* < 0.001; females: β = 0.44, *p* < 0.001), with both HAB and RM mediating the TA-CRA relationship in males, while only HAB served as a significant mediator in females. These findings advance our theoretical understanding of sex-specific aggression mechanisms in digital contexts and suggest that intervention strategies should be tailored differently for male and female college students.

## 1 Introduction

In recent years, the growing problem of cyber aggression has emerged as a significant concern within the realm of child and adolescent development (Peterson and Densley, 2017). This form of interpersonal aggression, which can manifest as cyberbullying, online harassment, or hate speech, has been shown to have negative consequences on the mental health and wellbeing of young individuals (Kowalski et al., 2014). A particularly vulnerable population in this context is college students, who are at an increased risk for both perpetrating and experiencing cyber aggression (Mishna et al., 2018). As digital connectivity becomes increasingly integrated into daily life, understanding the prevalence and related factors of cyber aggression among this group is imperative in order to develop effective interventions and policies to mitigate its harmful effects (Nixon, 2014).

### 1.1 Trait anger, hostile attribution bias, and revenge motivation in CRA

Cyber reactive aggression (CRA) is characterized by immediate, emotionally driven responses to specific incidents in the online realm. Investigating CRA is distinctively important because it often serves as the precursor to more sustained forms of conflict; a single reactive instance can escalate into a “flame war” or prolonged harassment. Furthermore, interventions designed for premeditated cyberbullying may be ineffective against the impulsive, heat-of-the-moment nature of CRA, necessitating a specific understanding of its unique psychological drivers.

Trait anger (TA) is a relatively stable disposition to experience anger frequently and intensely (DeWall et al., 2011). In digital interactions, higher TA is expected to increase the likelihood of anger activation when encountering perceived provocation, thereby elevating risk for impulsive retaliatory responses characteristic of CRA. Hostile attribution bias (HAB) refers to the tendency to interpret ambiguous social cues as hostile (Gagnon et al., 2017). Because online messages often lack tone, facial expressions, and other contextual cues, ambiguity is common in computer-mediated communication, which may increase the likelihood that individuals high in HAB infer hostile intent and respond reactively (Pettalia et al., 2013). Revenge motivation (RM) reflects a desire to retaliate following perceived wrongdoing (Quintana-Orts et al., 2020). In online settings, RM may be especially consequential because digital platforms provide fast and low-effort opportunities to respond, potentially facilitating immediate retaliation and escalation (Bushman et al., 2001; Hsieh, 2020; Véliz, 2019).

Several features of online communication may amplify the pathway from these psychological vulnerabilities to CRA. The online disinhibition effect and perceived anonymity can reduce accountability and normative constraints, lowering thresholds for aggressive responding (Suler, 2004). Meanwhile, the limited availability of nonverbal and situational cues can increase interpretive uncertainty, which may strengthen hostile inferences among individuals prone to HAB (Pettalia et al., 2013). Together, elevated anger reactivity (TA), hostile interpretation under ambiguity (HAB), and retaliatory decision tendencies (RM) may jointly contribute to CRA, which is defined by rapid, emotionally driven aggression in response to perceived provocation in digital settings (Runions, 2013).

These mechanisms can be organized within the General Aggression Model (GAM; DeWall et al., 2011). In GAM terms, trait anger functions primarily as a relatively stable person input that increases the likelihood of anger-related internal states when provoked online. Hostile attribution bias reflects a cognitive route mechanism that shapes how ambiguous online cues are interpreted, and revenge motivation captures a more proximal appraisal/decision tendency toward retaliation. Cyber reactive aggression represents the behavioral outcome of these processes in digital settings. This process framing clarifies why examining TA, HAB, and RM jointly can inform theory beyond documenting bivariate associations.

### 1.2 Sex differences in these psychological processes

Traditional literature has often characterized males as more prone to overt aggression and females to relational or social forms of aggression (Björkqvist, 2018; Yan et al., 2024). However, this binary perspective oversimplifies the complex nature of gendered behavior. Contemporary research emphasizes the need to recognize overlaps, individual variations within each sex, and the significant influence of social and cultural factors on aggressive behaviors (Eisner and Malti, 2015). While males tend to more frequently engage in direct, overt aggression, such as verbal insults or confrontations, females also participate in similar behaviors, especially in online interactions where anonymity can lower inhibitions (Fichman and Sanfilippo, 2015). Similarly, relational aggression, typically associated with females, such as social exclusion or spreading rumors, is also present among male students. The digital landscape facilitates various forms of aggression that are not strictly confined to gendered expectations (Crick and Grotpeter, 1995).

Moreover, males and females may differ, on average, in their motivations for engaging in CRA. Males might be more inclined toward retaliatory aggression driven by anger and the desire to assert dominance, while females might more often engage in aggression as a means of social manipulation or to protect social networks (Card et al., 2008). Additionally, the expression of aggression can be influenced by the type of online platform. For example, social media environments that emphasize visibility and peer feedback may encourage different aggressive behaviors compared to more anonymous platforms like forums or gaming communities (Fox and Moreland, 2015).

In addition, while males generally exhibit higher levels of overt cyber aggression, females are increasingly participating in direct forms of aggression facilitated by the anonymity of online platforms (Festl and Quandt, 2016). This shift suggests that the digital environment can mitigate some traditional gender barriers, allowing for more varied expressions of aggression among females. Research also indicates that females may experience and respond to online provocations differently, often seeking social support or reconciliation. However, when driven by high trait anger or revenge motivation, females can engage in aggressive behaviors akin to their male counterparts (Zajenkowska and Rajchert, 2020).

The interaction between sex and psychological factors such as TA, HAB, and RM further complicates the landscape of CRA. For example, while both sexes with high TA are prone to aggression, males may express this anger more outwardly online, whereas females might internalize it unless specific triggers amplify their reactive aggression (DeWall et al., 2011). Males with high HAB may more readily interpret ambiguous online cues as hostile, leading to immediate overt aggression, whereas females might display HAB in more relational or covert forms unless the context strongly provokes a retaliatory response (Dodge, 2006). Similarly, both sexes with high RM can engage in CRA, but the manifestations may differ. Males might prefer direct retaliation, such as public shaming, while females might engage in more nuanced forms of revenge that can include both direct and relational aggression (Quintana-Orts et al., 2020).

### 1.3 The present study

Guided by the GAM, we examine whether CRA is linked to anger-related input (TA), cognitive interpretation processes under ambiguity (HAB), and retaliatory decision tendencies (RM), and whether the organization of these links differs by sex. Thus, our focus is not only whether TA relates to CRA, but whether sex-differentiated route and appraisal/decision patterns characterize CRA. The focus on college students is particularly relevant given their extensive engagement with digital platforms and the heightened risk of both perpetrating and experiencing cyber aggression within this demographic. TA is posited as the exogenous variable in our model, given its established link to aggressive behaviors. Individuals with higher levels of TA are more likely to perceive and respond aggressively to online provocations. HAB serves as a mediating variable, where individuals who frequently interpret ambiguous online cues as hostile are more prone to engage in reactive aggression. RM is considered both a mediator and an outcome variable, representing the desire to retaliate against perceived transgressors, which can escalate aggressive interactions in digital environments.

This study employs network analysis and structural equation modeling (SEM) to explore the intricate pathways between TA, HAB, RM, and CRA. By incorporating sex as a moderating variable, we aim to identify how these relationships may differ between male and female college students. Previous research suggests that males and females may exhibit distinct patterns of aggression, influenced by both psychological predispositions and the unique dynamics of online interactions. Understanding the sex-specific pathways that lead to CRA can provide valuable insights for developing targeted prevention and intervention programs aimed at reducing cyber aggression and promoting healthier online interactions. We propose the following hypotheses: (1) Hypothesis 1: TA will positively predict CRA in both sexes, serving as the primary exogenous variable; (2) Hypothesis 2: HAB and RM will mediate the relationship between TA and CRA; (3) Hypothesis 3: Distinct sex-specific pathways will emerge.

## 2 Method

### 2.1 Participants and procedure

Nine hundred and twenty-six college students (aged 16–23 years, *M* = 18.58, SD = 0.96) were recruited through convenience sampling from twelve universities in China, including Heilongjiang, Anhui, Fujian and Guangzhou provinces, and asked to complete a series of questionnaires. While this sampling method allowed for efficient data collection across multiple provinces, it limits the generalizability of the findings. As participants self-selected into the study, there is a potential for selection bias. College students were invited to participate in the self-report survey anonymously which data were collected by means of an online survey. We sent the participants an online link to the survey which contained the consent first and then scales, and assured them of the strict confidentiality of the collected data. All participants who completed the survey were invited to enter a draw to win a random red envelope. This study was approved by the Ethics Committee of the authors' institute.

Basic demographic information, such as sex, age, whether the participants had left-behind experiences, and parents‘ education levels (ranging from primary school to master's degree and above), was collected for each college student. Detailed demographic characteristics of the sample are presented in Table 1. In this study, we operationalized “sex” as a binary variable (male/female) based on biological sex assignment at birth. Given the exploratory nature of sex-specific mechanisms in this specific cultural context, a binary framework was utilized to establish baseline differences. Consequently, the term “sex” is used throughout the manuscript to denote this binary classification. The “left-behind” phenomenon is increasingly relevant in China, largely due to significant rural-to-urban migration. These children, often cared for by relatives or left alone, form a distinct social group, with over 41 million in rural areas as of 2020 (National Bureau of Statistics United Nations Children's Fund, 2023). These children may have adverse experiences with an increased risk of behavioral issues, including cyber aggression (Wang et al., 2024). The transition to a digital-centric environment in higher education may further influence these individuals' online behaviors, particularly in the context of cyber reactive aggression.

**Table 1:** Descriptive statistics for the measurements on 926 college students.

**Variables**	**TA**		**HAB**		**RM**		**CRA**	
	***M***	***SD***	***M***	***SD***	***M***	***SD***	***M***	***SD***
**Sex**								
Female participants (*N* = 555)	1.81	0.44	2.54	1.06	2.85	1.03	1.19	0.30
Male participants (*N* = 371)	1.67	0.45	2.33	1.09	2.58	1.04	1.23	0.30
**Left-behind experience**								
Left-behind (*N* = 105)	1.89	0.42	3.04	0.90	3.10	1.11	1.29	0.26
No-left-behind (*N* = 821)	1.74	0.44	2.38	1.08	2.69	1.02	1.20	0.30
**Father education**								
Primary (*N* = 198)	1.80	0.46	2.40	1.11	2.91	0.94	1.22	0.32
Junior high (*N* = 409)	1.73	0.46	2.39	1.00	2.63	0.99	1.17	0.26
High school (*N*= 214)	1.71	0.40	2.48	1.22	2.81	1.24	1.25	0.37
College (*N*= 99)	1.85	0.36	2.72	0.98	2.72	0.94	1.20	0.23
Master's or above (*N*= 6)	1.73	0.21	3.40	0.16	3.00	0.00	1.40	0.13
**Mother education**								
Primary (*N* =317)	1.73	0.48	2.22	1.01	2.66	0.93	1.12	0.20
Junior high (*N* = 367)	1.73	0.40	2.50	1.06	2.71	1.04	1.21	0.31
High school (*N* = 171)	1.81	0.43	2.82	1.21	3.06	1.12	1.35	0.39
College (*N* = 67)	1.85	0.47	2.31	0.84	2.49	1.20	1.20	0.24
Master's or above (*N* = 4)	1.60	0.01	3.50	0.00	3.00	0.01	1.31	0.00

### 2.2 Measures

The Chinese version of the Trait Anger Scale was used to measure trait anger. The validity of the scale has been confirmed in Chinese research (Luo et al., 2011), and the current study found it to have high reliability (Cronbach's alpha = 0.86). The scale consists of 10 items rated on a 4-point Likert scale ranging from 1 (*almost never*) to 4 (*almost always*). Item scores are averaged to produce a total score, with higher scores indicating a higher level of Trait Anger.

The Word Sentence Association Paradigm for Hostility Scale, consisting of 16 situational sentences, was used to measure HAB (Beard and Amir, 2009). The scale has been translated to Chinese and demonstrated adequate psychometric properties (Quan and Xia, 2019). In the current study, the scale obtained a high Cronbach's alpha of 0.95. For each ambiguous scenario, participants rated the relatedness of a hostile word on a 6-point scale ranging from 1 (*not at all related*) to 6 (*very much related*). Higher averaged scores represent a stronger Hostile Attribution Bias.

The Revenge Motivation Scale was assessed using the subscale of the Transgression-Related Interpersonal Motivations Inventory (TRIM). The scale has demonstrated high reliability and validity with Chinese college students (Quan and Xia, 2019), and obtained a Cronbach's alpha of 0.95 in the current study. This 5-item subscale utilizes a 5-point Likert scale ranging from 1 (*strongly disagree*) to 5 (*strongly agree*). Higher averaged scores indicate a higher level of revenge motivation.

The CRA subscale of the Adolescent Online Aggressive Behavior Scale was used to measure CRA. The scale has been widely used among Chinese adolescents with acceptable validity and reliability (Tong-Lin et al., 2019), and in the current study, it demonstrated high reliability with a Cronbach's alpha of 0.90. Participants rated items regarding their online behavior on a 4-point scale ranging from 1 (*never*) to 4 (*always*). Higher averaged scores correspond to higher levels of Cyber-Reactive Aggression.

### 2.3 Network analysis and structural equation model

Network analyses with a frequency variable were conducted in R using the *mgm* package, since *mgm* allows for the explicit estimation of graphical models with mixed variable types (Haslbeck and Waldorp, 2015). These parameters were specified in the *mgm* function as lambdaGam = 0.25 and alphaGam = 0.25 (Ruan et al., 2023). These tuning parameters were selected to control the sparsity of the network using the Extended Bayesian Information Criterion (EBIC). Specifically, the gamma hyperparameter (0.25) was chosen to minimize the inclusion of spurious edges (false positives) while maintaining sensitivity to true associations. In *mgm*, the *glmnet* package was used to provide regression analysis with L1 and/or L2 regularization. To further validate the results of the network analysis and examine the consistency of the model across sex, we utilized the “lavaan” package (version 0.69) to construct multi-group SEM. In our research, the SEM model was primarily utilized for group comparisons, with parents' education levels and left-behind experiences as the covariates. Latent variables, typically represented by elliptical wireframes, were not employed in this basic model structure. Given that our basic model is a saturated model (due to the exclusive use of manifest variables), traditional global model fit indices such as RMSEA, CFI, TLI, and SRMR were not applicable and hence not reported. In the subsequent stages of our analysis, unsaturated models were developed by introducing constraints to the basic model. This modification led to non-saturated structures where assessing model fit becomes relevant.

## 3 Results

### 3.1 Descriptive statistics

Descriptive statistics for the measurements on 926 college students are found in Table 1.

### 3.2 Network analysis

**Figure 1:**
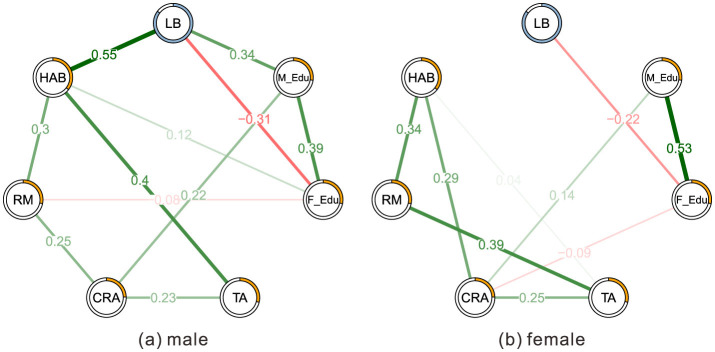
Two estimated MGM network structures based on 371 males **(a)** and 555 female participants **(b)**. The networks show the relationships among variables. The edge weights are the regression coefficients with regularization. Green edges represent positive relationships and red edges indicate negative relationships. The thickness of the edge reflects the magnitude of the relationship.

In the reported network models, edges represent partial correlation coefficients between variables after controlling for all other nodes in the network. According to the network analysis results, we can see the differences between male and female participants (see Figure 1):

(1) Male participants' HAB has a high positive association with TA (weight = 0.4), while female participants do not have such an association.
(2) Male participants' HAB has no association with CRA, while female participants' HAB and CRA are closely related.
(3) Male participants' RM and CRA (weight = 0.25) are related, while female participants do not have this relationship.
(4) Mothers' education levels are positively associated with aggressive behavior (weight = 0.22 for male and 0.14 for female participants).
(5) For male participants, left-behind experience is associated higher HAB (Hostile attribution bias) (weight = 0.55), while female participants didn't have such an association. In addition, left-behind participants‘ fathers have lower education levels (weight = −0.31 for male and −0.22 for female participants). However, for males, left-behind experience is associated with higher mothers' education levels (weight = 0.34).

### 3.3 Structural equation model

**Figure 2:**
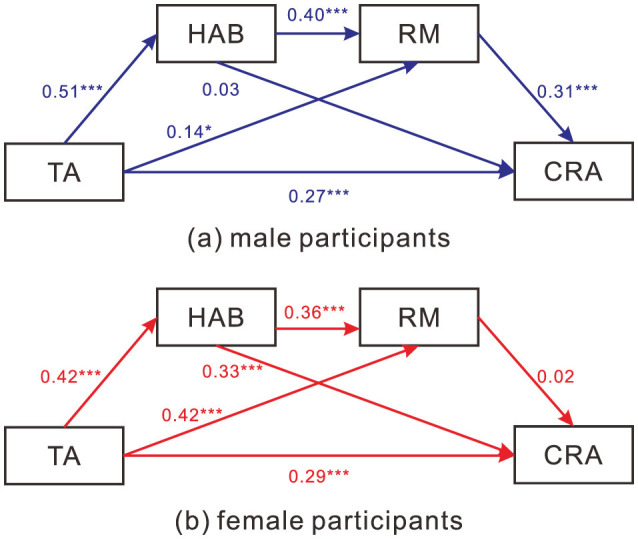
The CRA structural path models for **(a)** male and **(b)** female participants. Note: * *p* < 0.05, ** *p* < 0.01, *** *p* < 0.001.

Free-estimation model is displayed in Figure 2. The results of the mediation analysis with free-estimation for the male and female cyber-attack models are presented in Tables 2-1, 2-2, respectively. In the male model, the total effect of TA on CRA was significant, indicating that male TA has a significant positive association with CRA. Moreover, two indirect effects and the direct effect were significant (*p* < 0.05), suggesting that in male, HAB and RM partially mediated the relationship between TA and CRA. For the female model, the total effect of TA on CRA was also significant. In this case, only the indirect effect of TA → HAB → CRA was significant (*p* < 0.001), indicating that in female, HAB partially mediated the relationship between TA and CRA.

**Table 2-1:** The mediation effects of the male participants' CRA model.

**Paths**	**Effects**	**SE**	***Z***	***p***	**Bootstrapping 95% CI**
**Indirect effect**					
TA → HAB → CRA	0.02	0.03	0.61	0.54	[−0.04, 0.08]
TA → RM → CRA	0.04	0.02	2.34	0.02	[0.01, 0.08]
TA → HAB → RM → CRA	0.07	0.02	4.52	< 0.001	[0.03, 0.11]
**Direct effect**					
TA → CRA	0.27	0.05	5.21	< 0.001	[0.17, 0.37]
Total effect	0.40	0.05	8.81	< 0.001	[0.30, 0.50]

**Table 2-2:** The mediation effects of the female participants' CRA model.

**Paths**	**Effects**	**SE**	***Z***	***p***	**Bootstrapping 95% CI**
**Indirect effect**					
TA → HAB → CRA	0.14	0.02	6.19	< 0.001	[0.10, 0.18]
TA → RM → CRA	0.01	0.02	0.50	0.61	[−0.03, 0.05]
TA → HAB → RM → CRA	0.004	0.01	0.50	0.61	[−0.02, 0.02]
**Direct effect**					
TA → CRA	0.29	0.05	6.54	< 0.001	[0.19, 0.39]
Total effect	0.44	0.04	11.28	< 0.001	[0.36, 0.52]

Model comparison results show significant changes in model fit after constraining invariance between groups, as shown in Table 3. Above results indicate that within the theoretical framework of this study, there are cross-sex differences in the mediation pathways of CRA. Critical ratios analysis revealed significant differences in path coefficients between sex, including higher path coefficient for male from RM to CRA, lower path coefficient for male from HAB to CRA, and significantly higher path coefficient of TA to RM in female compared to male. Moreover, female did not show a significant association with from RM to CRA, while no significant effect was found on HAB to CRA in male (see Table 4).

**Table 3:** Model comparison of SEM for male and female participants.

**Fit indices**	**χ^2^**	**Df**	**Δχ^2^**	***p***	**RMSEA**	**CFI**	**NFI**
Free-estimation model (Model 1)	(–)	(–)	(–)	(–)	0	1	1
Intercept-invariant model (Model 2)	5.30	3	5.30	0.15	0.04	0.998	0.995
Constrained path coefficients model (Model 3)	56.28	9	56.28	< 0.001	0.107	0.955	0.948

Model comparisons in the table are based on results with the free estimation model (Model 1) as the reference.

**Table 4:** Multi-group critical ratio analysis.

**Path coefficients**	**Female participants**	**Male participants**	**Critical ratio**
TA → HAB	0.42^***^	0.51^***^	1.21
TA → RM	0.42^***^	0.13^*^	3.23^*^
TA → CRA	0.29^***^	0.27^***^	1.07
HAB → CRA	0.33^***^	0.03	11.00^**^
RM → CRA	0.02	0.31^***^	15.50^***^
HAB → RM	0.36^***^	0.40^***^	1.11

^*^p < 0.05, ^**^p < 0.01, ^***^p < 0.001.

## 4 Discussion

The present study aimed to investigate sex differences in the psychological processes of CRA, focusing on the roles of TA, HAB, and RM as mediators. The results from both network analysis and SEM provided valuable insights into the complex relationships among these variables and highlighted significant sex differences in the mediation pathways of CRA. Importantly, because our multi-group SEM relied on manifest composite scores rather than latent factors, the observed sex differences in path coefficients should be interpreted as preliminary, as they may be influenced by unmodeled measurement error and potential measurement non-equivalence across sex.

Similarities between sex were observed in the correlation between TA and CRA. Network analysis also showed the significant relationship between TA and CRA in both male and female participants. This finding is consistent with previous research that has highlighted the importance of TA as a key predictor of aggressive behavior, both online and offline (Sest and March, 2017; Yang J. et al., 2021). The current study extends this knowledge by demonstrating the relevance of TA in the context of CRA, suggesting that interventions targeting the reduction of TA might be effective in reducing aggressive behavior in cyberspace for both male and female participants.

Despite these similarities, there were also substantial differences in the mediating pathways between the sex. The network analysis results demonstrated that male participants' HAB has a strong positive association with TA, while no such association was found among female participants. However, SEM doesn't show differences in TA-HAB relationships between sex, with critical ratio insignificant. A notable divergence emerged regarding the TA-HAB relationship. Network analysis identified a strong edge for males but not females, whereas SEM indicated significant paths for both. This discrepancy likely stems from the methodological differences: the MGM network analysis employs L1-regularization (LASSO) to shrink partial correlations, often reducing redundant or weaker associations to zero to produce a sparse graph. In contrast, SEM estimates parameters based on the full covariance matrix without this aggressive shrinkage. Consequently, while the SEM results suggest the TA-HAB link exists for females, the network analysis implies it may be less robust or redundant relative to other variables in the female network compared to the male network.

In addition, male participants' HAB had no association with CRA, while female participants' HAB and CRA were closely related. This finding was further supported by the SEM analysis, which indicated that HAB was a significant mediator for female participants but not for males. These findings indicate that HAB plays a differential role in CRA between male and female participants, with a more pronounced role in female participants. This is in line with previous research that has suggested that female participants may be more prone to engaging in relational aggression, which is characterized by manipulation and harming others through social exclusion (Crick et al., 2006; Crick and Grotpeter, 1995). The association between HAB and relational aggression has been well-established (Martinelli et al., 2018), and the current findings suggest that this relationship may extend to the realm of CRA as well. Similarly, RM has strong associations with CRA for males but not for female participants.

Based on the whole structures on the indirect path from TA to CRA, we found that male participants may be more likely to engage in CRA as a result of their higher levels of HAB and RM, while female participants' CRA may be primarily driven by their HAB. More specifically, RM played a more prominent role in the relationship between TA and CRA for male participants compared to female ones. This finding is consistent with previous research suggesting that males tend to be likely to engage in direct and overt forms of aggression in response to perceived provocations (Archer and Coyne, 2005; Crick and Grotpeter, 1995). In contrast, female participants may be relatively more inclined to engage in indirect forms of aggression, such as spreading rumors or excluding others from social groups, which may not involve the same level of RM (Crick et al., 2006; Crick and Grotpeter, 1995). This highlights the importance of considering sex-specific mechanisms in understanding the relationship between TA and CRA. Because all variables were assessed at a single time point, the indirect effects tested here should be interpreted as statistical mediation patterns (i.e., decomposition of associations) rather than evidence of temporal or causal mediation.

Our study found that for male participants, left-behind experience is associated with HAB (weight = 0.55), while female participants didn't show such an association. This aligns with recent research by Yang B. et al. (2021), which suggests that male left-behind children may be more prone to externalizing behaviors, including aggression, as a coping mechanism. Female left-behind children, on the other hand, might be more likely to internalize their distress, potentially leading to different manifestations of online behavior. This may be explained by that socialization patterns and emotional regulation strategies may differ significantly between sexes (Wood and Eagly, 2012). In Chinese society, boys are often socialized to be strong, assertive, and to suppress emotional vulnerability (Yang et al., 2024). When faced with the emotional challenges of being left behind, they may lack the tools to process these feelings constructively, leading to a higher likelihood of developing HAB and engaging in aggressive behaviors online. This aligns with the social learning theory perspective, suggesting that boys may learn to use aggression as a means of coping with stress and asserting dominance in social situations (Cohn and Zeichner, 2006). Additionally, the unique social and emotional isolation experienced by left-behind children—often characterized by prolonged separation from parents and inadequate emotional support—can lead to heightened emotional dysregulation, which may exacerbate aggression in digital environments. For males, this dysregulation may manifest as overt hostility and revenge motivation, driving cyber reactive aggression (CRA). In contrast, for females, the emotional impact of being left behind may manifest in subtler, more socially mediated forms of aggression, such as rumor-spreading or cyber ostracism, which are not as directly linked to hostile attributions but are nonetheless potent forms of CRA (Festl and Quandt, 2016).

Associations involving demographic covariates (maternal education and left-behind experience) should be interpreted cautiously. These variables were included as contextual correlates rather than as focal constructs in our theoretical model, and the present design does not allow us to test the mechanisms that might explain these links. Therefore, the observed positive association between maternal education and CRA in both sexes is best considered exploratory and hypothesis-generating. One possibility is that maternal education captures unmeasured contextual factors (e.g., family socioeconomic resources, parental expectations, time availability for supervision, or parenting practices) that may relate to online behavior; however, these mechanisms were not directly assessed in the current study. Similarly, the finding that left-behind experience was associated with higher HAB among males (but not females) is an exploratory sex-specific correlate. Although prior work suggests that left-behind experiences may be linked to socioemotional adjustment and externalizing behaviors, our data do not permit conclusions about developmental pathways. Future research should measure parenting processes, peer contexts, and emotion-regulation development to test whether and how left-behind experiences shape hostile interpretations in digital interactions.

### 4.1 Cultural considerations

We acknowledge that culturally specific dynamics are likely pivotal in shaping the mechanisms of CRA within this population. One important cultural factor is the phenomenon of “left-behind” children, which is more prevalent in rural China due to significant rural-to-urban migration (Ruan et al., 2024; Wen et al., 2021). We propose that culturally specific dynamics may shape the mechanisms of CRA, though these interpretations remain exploratory. The observed association between left-behind experience and HAB in males might hypothetically reflect how early social isolation interacts with gender socialization, potentially fostering externalizing coping mechanisms—a pattern consistent with research linking isolation to aggression (Brandt et al., 2022). Furthermore, the positive association between maternal education and CRA could be hypothesized to reflect unmeasured pressures, such as high academic expectations, which have been linked to behavioral issues in similar contexts (Lee et al., 2021). Finally, cultural values such as collectivism and face-saving may also influence these expressions of aggression, warranting future investigation (Crossler et al., 2019; Wright et al., 2018).

### 4.2 Practical implications

These findings point toward tailored intervention strategies for college counseling centers. For male students, interventions might be most effective if they target the “anger-to-revenge” pathway. Techniques such as impulse control training and “cooling down” periods before responding to online stimuli could disrupt the link between Trait Anger and Revenge Motivation. Conversely, for female students, where the pathway is predominantly cognitive (via HAB), interventions should focus on cognitive restructuring and social information processing. Workshops that help female students generate alternative, non-hostile explanations for ambiguous online messages could reduce the likelihood of reactive aggression.

### 4.3 Limitations

The present study has several limitations that dictate specific directions for future research. First, while SEM enables the examination of complex relationships, our reliance on cross-sectional data prevents definitive conclusions about cause-and-effect relationships. Consequently, the mediation pathways identified here should be interpreted as statistical associations; future studies should employ longitudinal cross-lagged panel designs to confirm whether TA and HAB temporally precede CRA. Second, the sample consisted solely of Chinese college students, which restricts the generalizability of our findings. Future research should replicate this model across diverse cultural contexts to examine if these sex-specific mechanisms hold globally. Third, relying solely on self-report measures for sensitive topics like aggression may introduce social desirability bias. To address this, future work could utilize experimental paradigms (e.g., simulated social media interactions) to observe behavioral aggression objectively. Finally, our SEM analysis used observed composite scores rather than latent measurement models. Future investigations should establish measurement invariance at the item level to ensure that the observed sex differences in aggression pathways are not artifacts of measurement error.

## 5 Conclusion

These findings suggest sex-differentiated association patterns among TA, HAB, RM, and CRA at the observed-score level. Through network analysis and SEM, we revealed complex relationships between psychological factors and CRA, uncovering significant sex-based variations. While trait anger was associated with CRA in both sexes, the mediating pathways differed substantially. For males, both hostile attribution bias and revenge motivation mediated this relationship, while for females, hostile attribution bias emerged as the primary mediator. These findings emphasize the need for sex-specific approaches when examining CRA mechanisms. Additionally, the impact of maternal education levels and left-behind experiences underscores the importance of considering demographic factors in understanding CRA. However, given the study's limitations regarding cross-sectional design and self-report measures, these findings should be interpreted cautiously.

## Data Availability

The data analyzed in this study is subject to the following licenses/restrictions. Data are available from the corresponding author upon reasonable request. Requests to access these datasets should be directed to Qian-Nan Ruan, ruanqiannan@foxmail.com.
